# Collisions in outer space produced an icosahedral phase in the Khatyrka meteorite never observed previously in the laboratory

**DOI:** 10.1038/srep38117

**Published:** 2016-12-08

**Authors:** Luca Bindi, Chaney Lin, Chi Ma, Paul J. Steinhardt

**Affiliations:** 1 Dipartimento di Scienze della Terra, Università di Firenze, Via La Pira 4, I-50121 Florence, Italy; 2 Department of Physics, Princeton University, Jadwin Hall, Princeton, NJ-08544, USA; 3 Division of Geological and Planetary Sciences, California Institute of Technology, Pasadena, CA-91125, USA; 4 Princeton Center for Theoretical Science, Princeton University, Princeton, NJ-08544, USA

## Abstract

We report the first occurrence of an icosahedral quasicrystal with composition Al_62.0(8)_Cu_31.2(8)_Fe_6.8(4)_, outside the measured equilibrium stability field at standard pressure of the previously reported Al-Cu-Fe quasicrystal (Al_*x*_Cu_*y*_Fe_*z*_, with *x* between 61 and 64, *y* between 24 and 26, *z* between 12 and 13%). The new icosahedral mineral formed naturally and was discovered in the Khatyrka meteorite, a recently described CV3 carbonaceous chondrite that experienced shock metamorphism, local melting (with conditions exceeding 5 GPa and 1,200 °C in some locations), and rapid cooling, all of which likely resulted from impact-induced shock in space. This is the first example of a quasicrystal composition discovered in nature prior to being synthesized in the laboratory. The new composition was found in a grain that has a separate metal assemblage containing icosahedrite (Al_63_Cu_24_Fe_13_), currently the only other known naturally occurring mineral with icosahedral symmetry (though the latter composition had already been observed in the laboratory prior to its discovery in nature). The chemistry of both the icosahedral phases was characterized by electron microprobe, and the rotational symmetry was confirmed by means of electron backscatter diffraction.

Quasicrystals^1^,^2^, short for quasiperiodic crystals, are solids able to violate the conventional rules of crystallography because their structure is “quasiperiodic” rather than periodic; that is, their atomic density can be described by a finite sum of periodic functions with periods whose ratio is irrational. Their diffraction pattern consists of true Bragg peaks whose positions can be expressed as integer linear combinations of D integer linearly independent wavevectors where D is greater than the number of space dimensions. Among the quasicrystals made in the laboratory, many exhibit a crystallographically forbidden, three-dimensional icosahedral symmetry defined by D = 6 integer linearly independent wavevectors.

The first quasicrystalline phase found in nature, icosahedrite Al_63_Cu_24_Fe_13_^3^,^4^, displayed a five-fold symmetry in two dimensions and icosahedral symmetry in three dimensions and was found in the Khatyrka meteorite, a CV3 carbonaceous chondrite^5^,^6^,^7^. The discovery represented a breakthrough in mineralogy and in condensed matter physics. Then, a second quasicrystal, decagonite Al_71_Ni_24_Fe_5_^8^,^9^, was found in the same meteorite, and it was the first mineral to exhibit the crystallographically forbidden decagonal symmetry. Both icosahedrite and decagonite, however, showed compositions matching those of synthetic quasicrystalline phases found earlier^10^,^11^ in the laboratory at standard pressure.

Here we report the first icosahedral quasicrystal discovered in nature prior to being synthesized in the laboratory. It belongs to the Al-Cu-Fe system and exhibits the composition Al_62.0(8)_Cu_31.2(8)_Fe_6.8(4)_, which is outside the measured equilibrium stability field at standard pressure of the previously reported Al-Cu-Fe quasicrystal^12^,^13^,^14^ (Al_x_Cu_y_Fe_z_, with x between 61 and 64, y between 24 and 26, z between 12 and 13%). The new icosahedral phase was found in one of the meteoritic fragments of the same Khatyrka meteorite recovered from an expedition to the Koryak Mountains in far eastern Russia in 2011^5^,^7^ as a result of a search for material that would provide information on the origin of icosahedrite, the first natural quasicrystal. The fragment is labeled Grain 126A to distinguish it from others of Grain 126. All recovered fragments of Khatyrka including Grain 126 have been shown to have CV3-like oxygen isotopic compositions^6^,^15^,^16^, confirming their common meteoritic origin.

Most of the Khatyrka meteoritic fragments display evidence of an impact shock that generated a heterogeneous distribution of pressures and temperatures in which some portions of the meteorite exceeded 5 GPa and 1200 °C^15^. Other fragments of Grain 126 have previously led to the discovery of novel phases, including the new polymorph of Al, steinhardtite^17^, as well as other new crystalline Al-Cu-Fe alloys^18^. Other phases found include ringwoodite, coesite, stishovite, magnetite, diopside, forsterite, clinoenstatite, sodalite, nepheline, pentlandite, Cu-bearing troilite, icosahedrite, khatyrkite (CuAl_2_), cupalite (CuAl), taenite, Al-bearing trevorite, and Al-bearing taenite^13^.

## Results

### Description of the sample

**Figure 1:**
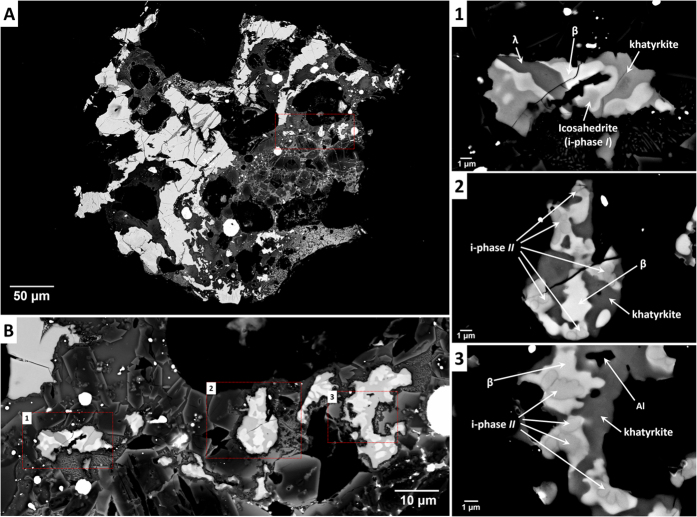
Backscattered electron images of Grain 126A. (**A**) Grain 126A; red dashed box indicates the region to be enlarged in (**B**). (**B**) The area where there are the three metal assemblages containing the two different icosahedral phases; red dashed boxes (indicated as 1, 2 and 3) indicate the regions to be enlarged in panels on right. Panels 1, 2 and 3 show the different associations of minerals in the three metal assemblages.

Grain 126A is dark grey in reflection under white incident light with visible shinier fragments reflecting the metallic constituents. Its small size – around ~0.4 mm in diameter –precludes determination of detailed optical properties of the minerals. A backscattered electron (BSE) image of 126A is shown in Fig. 1A. The two primary constituents of Grain 126A are: (1) large, disconnected Al-Cu-Fe metal assemblages (lighter, Fig. 1A); and, intimately surrounding these metal assemblages, (2) matrix material consisting of silicate glass and crystals of olivine and spinel (darker, Fig. 1A). Averaged and representative analyses of the different metal phases are presented in Table 1.

### Analysis of selected Al-Cu-Fe fragments

Al-Cu-Fe metals occur as small grains (Fig. 1B), generally irregular in shape and never spherical. They have a cuspate-lobate morphology, with cusps tending to point into the metal grains. The large Al-Cu-Fe metal fragments mostly comprise khatyrkite (CuAl_2_, with up to 2.68 elemental weight % Fe) and variable amounts of an unnamed (Al,Cu)Fe phase^18^ corresponding to the *β* phase in the Al-Cu-Fe system^14^,^19^. The *β* phase in 126A has the compositional range Al_55–60_Cu_38–43_Fe_1–3_, which overlaps with that of a known synthetic analogue. In addition to the *β* phase, the metal regions contain other previously unobserved Al-Cu-Fe phases. One is an unnamed phase with composition Al_73_Fe_19_Cu_8_, ideally Al_3_(Fe,Cu)^18^, corresponding to the *λ* phase in the Al-Cu-Fe system^19^.

Immediately adjacent to the *λ* grain are icosahedrite grains (denoted ‘i-phase *I*’). There are other metal grains that bear the same icosahedral quasicrystalline symmetry as icosahedrite but have a composition Al_62.0(8)_Cu_31.2(8)_Fe_6.8(4)_ (denoted ‘i-phase *II*’), which is significantly outside the measured equilibrium stability field of icosahedrite^12^,^13^,^14^ (Al_*x*_Cu_*y*_Fe_*z*_, with *x* between 61 and 64, *y* between 24 and 26, *z* between 12 and 13%), at standard pressure, even at temperatures up to 740 °C. Like *β* and *λ*, i-phase *II* is a previously unobserved phase. The i-phase *II* grains are completely enveloped by the *β* phase. The metal assemblages containing the i-phase *II* grains also have khatyrkite and metallic Al with a composition Al_97–98_Cu_2–3_.

**Figure 2:**
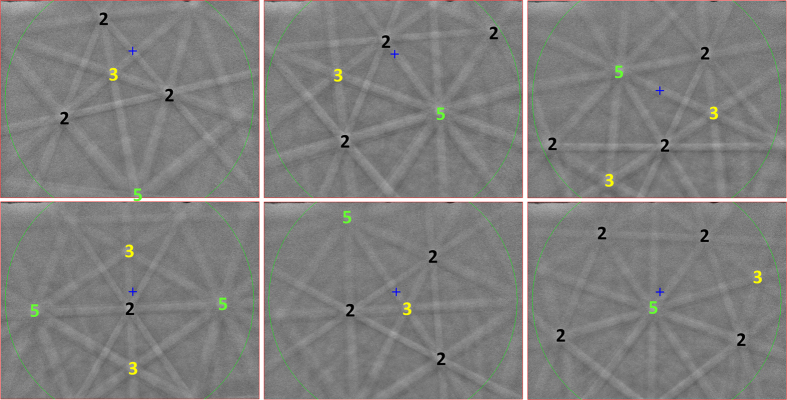
Electron backscatter diffraction patterns of the two icosahedral phases (the three patterns in the first row are from i-phase *I*, whereas those in the second row are from i-phase *II*) obtained from the regions labeled as i-phases in Fig. 1. The patterns match those predicted for a face-centered icosahedral quasicrystal.

Both i-phase *I* and i-phase *II* were studied by means of electron backscattered diffraction (EBSD). The EBSD patterns obtained (Fig. 2) display a pentagonal symmetry in their Kikuchi patterns. The sharpness of the Kikuchi bands indicates the quasicrystals have a well-ordered structure with icosahedral symmetry, containing at most minor structural defects. As expected, the EBSD software, which is designed to index crystalline phases, was unable to index this pattern in any standard crystal system.

## Discussion

The discovery of an icosahedral Al-Cu-Fe quasicrystal with a composition far from that of any known ideal, stable quasicrystal is notable for several reasons. It is only the third example of a natural quasicrystal to be found anywhere, all from fragments of the same Khatyrka meteorite; and it is the first documented example of the coexistence of two different Al-Cu-Fe i-phase compositions. Furthermore, it is the first example of a composition discovered in nature prior to being discovered in the laboratory.

**Figure 3:**
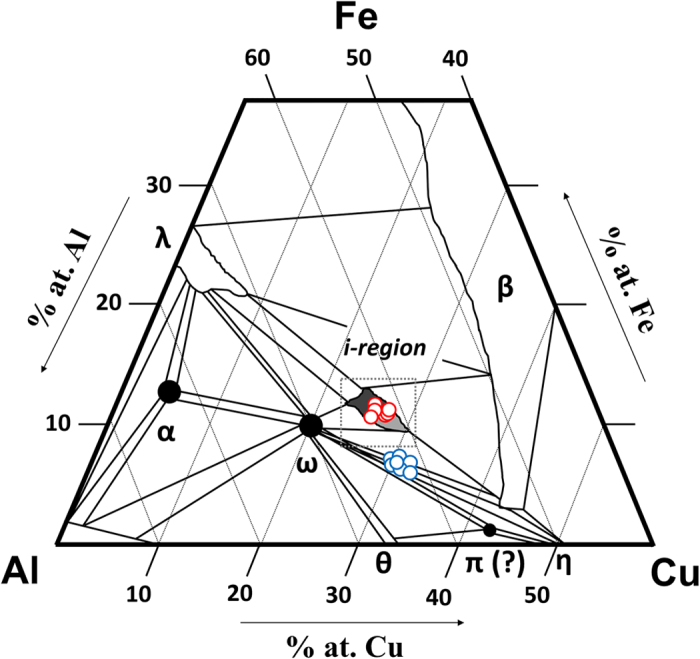
Subsolidus projection of the ternary Al-Cu-Fe phase diagram in the vicinity of the icosahedral phase (modified after Bancel 12). Shaded regions indicate pure phase fields and tie lines bound two-phase regions. The maximal extent of the icosahedral phase occurs within the boxed area labelled i-region. The dark shading shows the section of the i-region in which transformations have been identified. Empty red and light blue spheres correspond to data from i-phase *I* (icosahedrite) and i-phase *II*, respectively. Errors within the size of the symbols.

The Al-Cu-Fe ternary phase diagram at standard pressure has been systematically studied around the icosahedral region^12^,^13^,^14^. Icosahedrite, Al_63_Cu_24_Fe_13_ or equivalently i-phase *I*, lies within the stability field for the icosahedral quasicrystal phase as measured at all temperatures below melting. By contrast, i-phase *II*, Al_62.0(8)_Cu_31.2(8)_Fe_6.8(4)_, has a chemical composition that lies significantly outside the stability field at standard pressure for all temperatures below melting, for example, outside the stability field at room temperature first reported by Bancel^12^ (dashed area in Fig. 3) as well as at elevated temperatures up to 740 °C. A composition closer to that of i-phase *II* described here was reported by Zhang *et al*.^14^,^19^ during investigations on the Al-Cu-Fe system with low Fe content starting from an alloy with composition Al_56.8_Cu_37_Fe_6.2_ and annealed at 660 °C. Based on scanning electron microscopy (SEM) and X-ray powder diffraction measurements (which are not as precise as the methods reported here), they claimed an icosahedral phase with composition Al_62.3_Cu_28.6_Fe_9.1_, with significantly higher percentage of Fe and lower Cu than observed here in i-phase *II*. Gratias *et al*.^20^ found that at 680 °C, the stability field of the i-phase extends approximately over a triangle with vertices (62.4, 24.4, 13.2), (65, 23, 12) and (61, 28.4, 10.6), in terms of (Al, Cu, Fe) atomic %. They reported that this region splits schematically into 3 fields: (*i*) the perfect icosahedral phase, which is stable down to the lowest possible annealing temperature where atomic diffusion is active in a tiny region of composition close to Al_62.3_Cu_24.9_Fe_12.8_; (*ii*) a well-defined periodic phase with rhombohedral structure, which transforms reversibly into the icosahedral phase near 710 °C in the lower part of the triangle; (*iii*) a complex region characterized by additional diffraction effects (peak broadening, line-shapes, etc.), which may correspond to various approximant structures closely related to the i-phase. However, none of these earlier studies found evidence of the i-phase *II* composition.

One possible explanation for why the i-phase *II* has not been found in earlier studies is that i-phase *II* is a kinetically stabilized composition, only preserved because of very rapid quench, and is thermodynamically unstable at any pressure and temperature. Previous investigations of Khatyrka samples^15^ have provided ample evidence that the meteorite experienced an impact-induced shock that generated a highly heterogeneous distribution of pressures and temperatures with conditions as high as 5 GPa and 1,200 °C, followed by rapid cooling. On this basis, the kinetic stabilization explanation is plausible. On the other hand, it is notable that fragments of 126A containing i-phase *I*, as illustrated in panel 1 in Fig. 1, have textures qualitatively similar to fragments with i-phase *II*, as shown in panels 2 and 3. The metallic phases observed with i-phase *I* appear to have formed according to a predictable solidification sequence along a liquid line of descent, largely consistent with the equilibrium phase diagram for Al-Cu-Fe, beginning with the formation of the *λ* phase and including the formation of an icosahedral phase with a composition in the equilibrium stability field at standard pressure. Based on their texture, the fragments containing i-phase *II* appear to have cooled at a similar rate but starting from a different initial composition, such that the solidification process began with i-phase *II* and skipped the *λ* phase. In that case, i-phase *II* could have a composition in a stability field for i-phase that has shifted or expanded due to the high *P-T* induced by the shock, and likely be a new high-pressure phase, crystallized from shock-induced melt under high pressure. This latter possibility could be explored in the laboratory by beginning with a liquid with the composition of i-phase *II* at high *P-T* and checking if the i-phase *II* solid forms when high pressure is maintained as the temperature decreases. Combining studies of natural quasicrystals, high-pressure diamond anvil experiments^21^,^22^, and laboratory shock experiments^23^, we plan to test this hypothesis and develop a better understanding of the kinetic and thermodynamic stability of quasicrystals.

## Methods

### Sample characterization techniques

The sample studied here (Grain 126A) was investigated by means of SEM (scanning electron microscopy), EBSD (electron backscatter diffraction), and EMP-WDS (electron microprobe, wavelength dispersive spectrometry) techniques.

### Scanning Electron Microscopy

The sample was impregnated with epoxy and mechanically polished in flowing water with Al_2_O_3_-abrasive papers down to 5 μm and loose diamond powder down to 0.25 μm, followed by three hours of vibrational polishing in colloidal silica (30 nm in dia.) solution. Carbon coat was applied for SEM, EDS and EMP analyses, then removed for EBSD studies. The sample was examined on the Caltech GPS ZEISS 1550VP field emission SEM equipped with an angle-sensitive back-scattered electron detector, a 80 mm^2^ active area Oxford X-Max Si-drift-detector EDS, and an HKL EBSD system. Imaging, mapping, semi-quantitative EDS analysis, and EBSD of the previously identified regions were conducted using the SmartSEM, the AZtec and the Channel 5 softwares. Analyses used 20 kV accelerating potential and a 120 μm field aperture in high current mode (~6 nA probe current) for EBSD analysis and 15 kV for EDS analysis.

### Electron microprobe

After location of the i-phases and identification of major and minor elements by SEM, three regions were re-analyzed for Al, Cu, Fe, Cr, Ni, Si, Mg and Ca using WDS on a five-spectrometer JEOL 8200 electron microprobe in the GPS analytical facility at Caltech. High spatial resolution was achieved using conditions of 12 kV, 5 nA, and a focused beam. Pure metal standards were used. Counting times were 20 s on-peak and 10 s each on high and low background positions. Data reduction used the CITZAF routine built into the *Probe for EPMA* software.

## Additional Information

**How to cite this article**: Bindi, L. *et al*. Collisions in outer space produced an icosahedral phase in the Khatyrka meteorite never observed previously in the laboratory. *Sci. Rep.* **6**, 38117; doi: 10.1038/srep38117 (2016).

**Publisher's note:** Springer Nature remains neutral with regard to jurisdictional claims in published maps and institutional affiliations.

## Figures and Tables

**Table 1:** Electron microprobe analyses (wt% of elements with their standard deviations in parenthesis) and atomic ratios of metal phases in 126A.

Phase	*n*	Al	Fe	Cu	Ni	Si	Mg	Ca	Cr	Total
khatyrkite	16	47.9(2)	0.5(1)	51.2(5)	b.d.l.	b.d.l.	b.d.l.	b.d.l.	b.d.l.	99.60
	9	48.0(3)	0.7(2)	51.7(5)	b.d.l.	b.d.l.	b.d.l.	b.d.l.	b.d.l.	100.40
	13	47.7(3)	0.6(1)	51.3(5)	b.d.l.	b.d.l.	b.d.l.	b.d.l.	b.d.l.	99.60
	9	48.0(4)	1.04(6)	51.0(5)	b.d.l.	0.06(2)	b.d.l.	b.d.l.	b.d.l.	100.10
	5	48.9(5)	1.4(1)	50.1(8)	b.d.l.	b.d.l.	b.d.l.	b.d.l.	b.d.l.	100.40
	8	48.2(2)	0.9(1)	51.0(5)	b.d.l.	b.d.l.	b.d.l.	b.d.l.	b.d.l.	100.10
	11	48.1(4)	1.3(2)	50.8(9)	b.d.l.	b.d.l.	b.d.l.	0.03(2)	b.d.l.	100.23
	2	47.8(2)	2.68(6)	49.31(3)	b.d.l.	0.16(4)	b.d.l.	0.03(2)	0.07(3)	100.05
	2	48.7(5)	2.10(3)	49.94(9)	b.d.l.	0.09(5)	0.1(1)	b.d.l.	b.d.l.	100.93
	2	48.42(5)	2.16(7)	49.4(9)	b.d.l.	0.10(2)	b.d.l.	0.05(2)	b.d.l.	100.13
	2	47.2(1)	1.6(2)	50.20(3)	b.d.l.	0.2(1)	b.d.l.	b.d.l.	b.d.l.	99.20
*β-*phase	3	38.7(2)	1.5(1)	59.5(6)	b.d.l.	b.d.l.	b.d.l.	0.03(2)	b.d.l.	99.73
	3	38.3(1)	2.7(3)	57.8(5)	b.d.l.	b.d.l.	b.d.l.	b.d.l.	0.05(3)	98.85
	2	35.9(4)	4.23(4)	61.2(4)	b.d.l.	b.d.l.	b.d.l.	b.d.l.	0.07(2)	101.40
	3	33.92(8)	3.1(1)	62.7(6)	b.d.l.	b.d.l.	b.d.l.	b.d.l.	0.05(1)	99.77
	2	35.80(5)	2.7(3)	60.8(1)	b.d.l.	b.d.l.	b.d.l.	0.04(1)	b.d.l.	99.34
	2	36.1(3)	2.73(9)	60.8(9)	b.d.l.	b.d.l.	b.d.l.	b.d.l.	b.d.l.	99.63
*λ-*phase	4	55.0(4)	30.4(6)	14.2(3)	b.d.l.	0.30(1)	b.d.l.	0.03(1)	0.16(3)	100.09
i-phase *I*	3	43.2(1)	15.0(5)	41.0(4)	b.d.l.	0.14(6)	b.d.l.	0.04(2)	0.14(1)	99.52
i-phase *II*	3	42.0(7)	9.4(9)	47.3(9)	b.d.l.	0.08(5)	0.04(6)	b.d.l.	0.09(3)	98.91
i-phase *II*	9	40.3(5)	9.2(4)	48.7(6)	b.d.l.	0.06(3)	0.06(6)	0.04(1)	0.11(2)	98.47
***Formulae on the basis of 100 atoms***										
khatyrkite		68.54	0.35	31.11	—	—	—	—	—	100.00
		68.29	0.48	31.23	—	—	—	—	—	100.00
		68.37	0.42	31.21	—	—	—	—	—	100.00
		68.36	0.72	30.84	—	0.08	—	—	—	100.00
		69.02	0.95	30.03	—	—	—	—	—	100.00
		68.57	0.62	30.81	—	—	—	—	—	100.00
		68.40	0.89	30.68	—	—	—	0.03	—	100.00
		68.05	1.84	29.81	—	0.22	—	0.03	0.05	100.00
		68.47	1.43	29.82	—	0.12	0.16	—	—	100.00
		68.61	1.48	29.72	—	0.14	—	0.05	—	100.00
		67.93	1.11	30.68	—	0.28	—	—	—	100.00
*β-*phase		59.81	1.12	39.04	—	—	—	0.03	—	100.00
		59.68	2.04	38.24	—	—	—	—	0.04	100.00
		56.12	3.20	40.62	—	—	—	—	0.06	100.00
		54.65	2.42	42.89	—	—	—	—	0.04	100.00
		56.87	2.08	41.01	—	—	—	0.04	—	100.00
		57.09	2.09	40.82	—	—	—	—	—	100.00
*λ-*phase		72.27	19.29	7.92	—	0.38	—	0.03	0.11	100.00
i-phase *I*		63.44	10.64	25.57	—	0.20	—	0.04	0.11	100.00
i-phase *II*		62.87	6.80	30.07	—	0.12	0.07	—	0.07	100.00
i-phase *II*		61.40	6.77	31.51	—	0.09	0.10	0.04	0.09	100.00

*n*: number of analyses included in average, n.a.: not analyzed, b.d.l.: below detection limits, 0.07 wt% Ni, 0.05% Si, 0.04% Mg, 0.03% Ca, 0.05% Cr.
